# Molecular mechanisms underlying inherited photoreceptor degeneration as targets for therapeutic intervention

**DOI:** 10.3389/fncel.2024.1343544

**Published:** 2024-02-02

**Authors:** Andrea Bighinati, Elisa Adani, Agnese Stanzani, Sara D’Alessandro, Valeria Marigo

**Affiliations:** ^1^Department of Life Sciences, University of Modena and Reggio Emilia, Modena, Italy; ^2^Center for Neuroscience and Neurotechnology, Modena, Italy

**Keywords:** oxidative stress, inflammation, cGMP, calcium, ER-stress, rhodopsin

## Abstract

Retinitis pigmentosa (RP) is a form of retinal degeneration characterized by primary degeneration of rod photoreceptors followed by a secondary cone loss that leads to vision impairment and finally blindness. This is a rare disease with mutations in several genes and high genetic heterogeneity. A challenging effort has been the characterization of the molecular mechanisms underlying photoreceptor cell death during the progression of the disease. Some of the cell death pathways have been identified and comprise stress events found in several neurodegenerative diseases such as oxidative stress, inflammation, calcium imbalance and endoplasmic reticulum stress. Other cell death mechanisms appear more relevant to photoreceptor cells, such as high levels of cGMP and metabolic changes. Here we review some of the cell death pathways characterized in the RP mutant retina and discuss preclinical studies of therapeutic approaches targeting the molecular outcomes that lead to photoreceptor cell demise.

## 1 Introduction

The human retina is part of the central nervous system (CNS). It includes light-sensitive cells, called photoreceptors, which synapse with interneurons to convey the visual information to ganglion cells, which in turn target the brain through the optic nerve. Retinal neurons can, in principle, remain alive and active life-long, nevertheless their functionality and viability can be compromised by a large number of diseases that lead to vision impairment or blindness. The diseases can have hereditary or multifactorial nature and can be caused by genetic or environmental insults (Duncan et al., 2018; Fleckenstein et al., 2021). In this review we will focus on inherited diseases leading to photoreceptor degeneration, specifically on retinitis pigmentosa (RP), that is a major cause of blindness in young adults and remains an unmet medical need, with most of the forms of RP still untreatable today (Kutluer et al., 2020). RP patients initially experience decreased visual acuity, constricted visual field (tunnel vision) and bone spicule pigmentation in the fundus of the eye. Photoreceptor degeneration, unfortunately, progresses to complete visual loss. We will discuss the molecular pathways activated in the degenerative process, aim at fostering the understanding of the underlying mechanisms and present the preclinical therapeutic approaches that have been developed to target the activated cell death pathways.

The degeneration and loss of photoreceptors during RP progression is widely studied and several molecular mechanisms have been uncovered in animal models, comprising *Drosophila melanogaster*, zebrafish, rodents, pigs, and dogs. The most studied animal models are two RP mice with spontaneous mutations in the *Pde6b* (*Phosphodiesterase 6b*) gene, named *rd1* and *rd10*, and genetically modified mice bearing dominant mutations in the *Rhodopsin* (*Rho*) gene. The unfeasibility of collecting biopsies from patients during the progression of the disease and the limited number of post-mortem specimens at ongoing degeneration stages hampered our knowledge on the degenerative mechanisms in the human retina. Nevertheless, progress in ophthalmology research based on high-resolution retinal imaging have been fundamental for natural history studies and for monitoring photoreceptor degenerative progression. These data encouraged research with mutant animals because they can offer, at least in part, appropriate experimental models for retinal degeneration. More recently, *in vitro* techniques for culture of human retinal organoids derived from patient cells are providing samples to address several open questions on the mechanisms activated in the diseased retina (O’Hara-Wright and Gonzalez-Cordero, 2020). However, few reports on pathophysiological characterization of human samples are available, thus here we will discuss molecular changes underlying photoreceptor degeneration, focusing specifically on inherited forms of retinal degeneration, based on studies performed on animal models of the disease.

Retinitis pigmentosa (RP) is a form of inherited retinal degeneration characterized by high genetic heterogeneity with more than 200 genes linked to the disease^1^ (Broadgate et al., 2017). RP can be inherited as an autosomal dominant or autosomal recessive or X-linked disorder. The most frequent mode of inheritance is the autosomal recessive mode with 62% of diagnosed patients, the prevalence of the autosomal dominant form is 24% and 14% of cases have a X-linked trait (Bravo-Gil et al., 2017). Besides the forms of RP commonly diagnosed by early adulthood, we need to mention a juvenile form of RP, called Leber congenital amaurosis (LCA), that is diagnosed in children. Finally, RP can also be found in syndromic inherited diseases, such as Usher syndrome, characterized by congenital hearing impairment and RP, and Bardet-Biedl syndrome, which is a genetically heterogeneous ciliopathy. The genetic heterogeneity is mirrored in the clinical picture, where variable progression of retinal degeneration can be portrayed, but a gene-phenotype correlation was rarely defined. Most of the disease-causing genes are expressed specifically in photoreceptors, mainly operating in phototransduction and other photoreceptor specific functions, but other genes are either not photoreceptor specific or even ubiquitously expressed, such as pre-mRNA splicing genes. Despite this high multiplicity of genes and functions, some common pathophysiological events occurring at the subcellular and molecular levels have been clarified. Among these, molecular determinants are oxidative stress, high levels of cyclic guanosine-3′,5′-monophosphate (cGMP), calcium ion overload and endoplasmic reticulum (ER) stress (Power et al., 2020). Furthermore, metabolic and molecular alterations as well as inflammation affect the histology and functionality of the degenerating retina.

The neuroanatomy of the retina had been illustrated by Ramón y Cajal who depicted the six major layers of the retina. The lamellar structure is well organized to favor the progression of the visual stimulus from photoreceptor cells, in the outermost retinal layer, to the optic nerve fiber layer composed by ganglion cell axons. This highly ordered architecture allows ophthalmologists and researchers to track the development of the disease by histological examination, that is now possible *in vivo* in patients and animal models by the OCT (optical coherence tomography) technique.

Morphology and metabolism of the retinal cells are affected by negative events triggered during retinal degeneration and, when monitored, they can depict the progression of the pathology. These alterations in the retinal tissue are known as “retinal remodeling”. Retinal remodeling proceeds with three steps of changes: neuronal cell death, cell migration and changes in neuronal circuitry (Marc et al., 2003). Glial hypertrophy, move of neuronal cell bodies to different layers with alteration of the lamination and neuronal rewiring distinguish retinal remodeling. Photoreceptor degeneration is, in fact, accompanied by changes in wiring of retinal neurons (Strettoi et al., 2003). While these changes appear subtle, characterization of retinal remodeling during the degeneration process can provide important information on window of opportunity for an efficacious therapeutic intervention.

Other detectable changes in the progression of photoreceptor degeneration are molecular outcomes activated in response to different types of stress that occur to photoreceptor cells undergoing cell death. In this review we dedicate specific attention to the most well characterized stress events in RP, such as oxidative stress, inflammation, high levels of cGMP, calcium ions imbalance and protein misfolding, that triggers ER-stress.

## 2 Oxidative stress

Oxidative stress results from an imbalance in production of reactive oxygen species (ROS) and antioxidant capacity of the cell. ROS are highly reactive chemical species that can modify DNA, proteins and lipids and thereby affect several cellular mechanisms. In fact, oxidative stress impacts crucial pathways such as inflammation, unfolded protein response (UPR), autophagy and mitophagy (Domènech and Marfany, 2020). Both endogenous and exogenous factors contribute to the generation of ROS inside the cell. ROS can derive from several intracellular enzymatic reactions with the main producer being the mitochondrial respiratory chain during the process of oxidative phosphorylation, whereas exogenous factors include, for example, UV-light radiation, cigarette smoking and environmental pollution (Ren and Léveillard, 2022).

To prevent ROS accumulation, cells activate different antioxidant mechanisms, involving non-enzymatic and enzymatic systems. The first comprises small molecules that directly interact with ROS and neutralize them by accepting or donating electrons, while the second system engages enzymatic antioxidants that gradually convert free radicals into hydrogen peroxide and water. Superoxide dismutase (SOD), catalase (CAT), glutathione peroxidase (GPx) and thioredoxin (TRX) are some of the main enzymes responsible for the defense against ROS (Ren and Léveillard, 2022). Importantly, the enzymatic antioxidant response is tightly regulated to maintain intracellular redox homeostasis and the major director is the transcription factor NRF2 (nuclear factor erythroid-2-related factor 2), that can activate gene expression of antioxidant enzymes upon stress stimuli. Besides, NRF2 is also involved in the regulation of other important cellular pathways, including metabolism and inflammation and is therefore a critical constituent in the transition from physiological to pathological conditions (He et al., 2020).

### 2.1 Molecular pathways associated to oxidative stress in retinal degeneration

**FIGURE 1:**
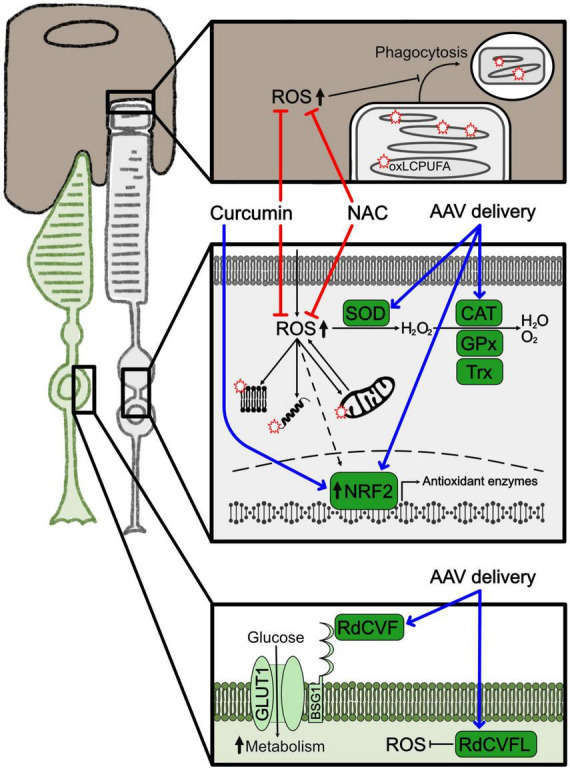
Oxidative stress and related treatments. Representation of the major molecular changes associated to oxidative stress. Cones are shown in green, rods in light gray and RPE in brown. Squares highlight cellular compartments where oxidative stress is activated. The upper square depicts the interface between RPE and the terminal part of the rod outer segment with the phagocytosis product; the square in the middle illustrates the cytoplasm of the rod photoreceptor with mitochondria, protein and lipids oxidized by ROS; the lower square shows the membrane and cytoplasm of a cone photoreceptor. Red stars represent peroxidation products. Blue arrows indicate overexpression of neuroprotective factors by gene therapy. Red lines indicate approaches to inhibit ROS production. Dashed arrows indicate indirect effects. AAV, adeno associated virus; CAT, catalase; GPx, glutathione peroxidase; NAC, N-acetylcysteine; oxLCPUFA, oxidized long chain polyunsaturated fatty acid; RdCVF, rod-derived cone viability factor; RdCVFL, rod-derived cone viability factor long; ROS, reactive oxygen species; SOD, superoxide dismutase; TRX, thioredoxin.

In general, the retina is highly vulnerable to oxidative stress because of the elevated presence of ROS (Domènech and Marfany, 2020). These are mainly generated by the high rate of oxygen consumption and the sustained metabolism of photoreceptor cells, together with the constant exposure to light. The photoreceptor outer segments are rich in long chain polyunsaturated fatty acids (LCPUFA) that are extremely susceptible to lipid peroxidation caused by ROS. Oxidized LCPUFA can significantly alter membrane composition of the cell and interfere with signaling pathways (Njie-Mbye et al., 2013). In addition, a direct consequence of LCPUFA oxidation is the accumulation of ROS also in the retinal pigment epithelium (RPE) due to the daily shedding of the photoreceptor outer segment (Figure 1). Nevertheless, the antioxidant systems can normally protect the retina from oxidative stress. In fact, in the degenerating retina of murine models of RP NRF2 was found upregulated in early phases of the disease (Comitato et al., 2020). Despite the presence of the antioxidant network, disruption of the cellular redox homeostasis plays a critical role in the progression of retinal degeneration and results in accumulation of ROS and consequent tissue damage (Batliwala et al., 2017). Furthermore, the ongoing degeneration of rods, that occurs during RP, results in oxygen accumulation, increased ROS production and progressive oxidative damage in surviving photoreceptor cells and other cell types, triggering additional pathways that altogether accelerate the progression of the disease (Gallenga et al., 2021). While oxidative stress is believed to play a significant role in rod photoreceptor cell demise, it seems to be the main cause of secondary cone degeneration (Shen et al., 2005; Komeima et al., 2006). Late stages of retinal degeneration have been suggested to be related to hyperoxia and related ROS damages that are the main contributors to cone death in RP. Based on these evidences, bolstering anti-oxidant response can preserve cone photoreceptors (Komeima et al., 2006; Usui et al., 2009).

A final aspect worth to mention is the link between oxidative stress and inflammation. Specifically, activation of microglia has been proposed to occur as response to oxidative stress stimuli establishing an unresolved cycle of inflammation in the retina (Gallenga et al., 2021). As previously reported, ROS oxidize lipids, proteins, and nucleic acid and, regarding the last, the most common modification occurs on guanine bases with the formation of 8-oxo-7,8-dihydro-2′-deoxyguanosine (8-oxoG). In proliferating cells, like microglia, incorporation of 8-oxoG in the genome induces base pair mismatches and eventually MutT homolog-1 (MTH1) activation, which mediates base excision repair (Nakatake et al., 2016). In animal models of RP oxidative stress markers were found accumulated in microglia cells that could themselves be a source of ROS. Under excessive oxidative stress conditions, MTH1 activity induces accumulation of single-strand breaks in DNA and activation of poly(ADP-ribose) polymerase (PARP) that significantly contributes to microgliosis and cell death (Murakami et al., 2020). The fact that elevated 8-oxoG was detected both in the retina of RP mice and in the vitreous of RP patients (Murakami et al., 2012) makes this pathway a relevant target to alleviate the retina from oxidative and inflammation damages during degeneration.

### 2.2 Therapeutic approaches to target oxidative stress

Activation of oxidative stress in photoreceptor degeneration is a common pathogenetic mechanism disregarding of the genetic variation. Therefore, antioxidants have been promoted as treatments to preserve photoreceptor function and survival (Figure 1; Campochiaro et al., 2015; Vingolo et al., 2022). Indeed, intake of different antioxidants provided a protective effect in animal models of RP (Komeima et al., 2006; Sanz et al., 2007; Zhang et al., 2022). Nonetheless, clinical trials based on supplementation of diverse antioxidants, like vitamin A, vitamin E, docosahexaenoic acid (DHA), and carotenoids, like lutein and 9-*cis*-β-carotenoid, conveyed contradictory results in RP patients (Berson et al., 1993, 2004, 2010; Hoffman et al., 2004; Bahrami et al., 2006; Rotenstreich et al., 2013). In fact, although the studies suggested an overall positive but limited capacity in delaying visual impairment, they failed to provide strong evidence for an antioxidant protection effect in the progression of the degeneration (Brito-García et al., 2017; Schwartz et al., 2020). Further research is needed to determine a safe and effective supplementation based on the combination of antioxidants that might be beneficial for specific subsets of RP patients (Comander et al., 2023). The antioxidant compound N-acetylcysteine (NAC) was shown to reduce oxidative stress in animal models (Lee et al., 2011; Yoshida et al., 2013) and improved visual functions of RP patients in a phase I clinical trial (Campochiaro et al., 2020). A new phase III clinical trial (NCT05537220) aims at testing the effects of oral administration of NAC to prevent secondary cone degeneration in a sustained treatment study.

Besides antioxidant molecules, modulation of the cellular enzymatic antioxidant response is another possible approach for delaying RP pathogenetic progression by targeting oxidative stress. Specifically, overexpression by AAV-based (adeno associated virus) delivery of proteins related to the NRF2 pathway, like OXR1 (oxidation resistance 1), NRF2 itself and the NRF2-induced enzymes SOD and CAT, was tested. These treatments significantly reduced oxidative stress in diverse cell types of the retina, including photoreceptors and RPE, and partially preserved visual function (Usui et al., 2009; Xiong et al., 2015; Sahu et al., 2021; Wu et al., 2021). Natural compounds are also able to upregulate the NRF2/enzymatic antioxidant pathway and were, thus, contemplated as possible therapeutic strategies because of the easier administration and higher availability compared to AAV-mediated gene therapy. For instance, curcumin is well known for its beneficial nutraceutical properties of mitigating oxidative stress and inflammation. Curcumin pleiotropic effects, that include upregulation of NRF2, increased antioxidant enzymes and reduced inflammatory mediators, could protect from degeneration models of retinal diseases, including RP (Vasireddy et al., 2011).

Another interesting new treatment to modulate the antioxidant response is the delivery of the rod-derived cone viability factor long (RdCVFL) to prevent secondary cone degeneration. This protein is encoded by the *Nucleoredoxin Like 1* (*NXNL1*) gene that, through alternative splicing, gives rise to a truncated isoform and a full-length isoform, RdCVF and RdCVFL, respectively. On one side, RdCVF is a neurotrophic factor secreted by rods and promotes cone survival by increasing glucose uptake and metabolism. On the other side, RdCVFL has thioredoxin activity and is involved in protection against oxidative stress (Byrne et al., 2015; Mei et al., 2016). Both proteins were shown to sustain cone viability and function with separate and complementary mechanisms. Oxidative stress damage is, in fact, also related to altered glucose metabolism and both these pathways together possibly contribute to cone cell death (Gallenga et al., 2021; Kanan et al., 2022). While an AAV-based gene therapy (SPVN06) to deliver these factors is currently investigated in a phase I/II clinical trial in RP patients, the dual role in photoreceptor neuroprotection of the *Nxnl1* gene, based on modulating the redox and the metabolic homeostasis, further underlines how elucidating the link between glucose metabolism and oxidative stress may open additional therapeutic opportunities to treat photoreceptor degeneration.

In summary, although there is a lot more to be explored about the mechanisms by which oxidative damage contributes to retinal degeneration, increased oxidative stress and decreased antioxidant capacity in retinal cells are negative events during the progression of RP. Moreover, although they are not the primary cause of the disease, these are common features regardless of the RP causative mutation. Therefore, the previously discussed treatment approaches or novel therapeutic targets may be relevant for the cure of a broad range of RP patients.

## 3 Inflammation

The CNS and the eye are privileged in terms of immunological and inflammatory responses compared to other organs. These specific features are maintained by the presence of barriers (e.g., blood-brain barrier and blood-retinal barrier, BRB), which exclude or limit the passage of circulating cells and substances to the CNS and the eye, hence keeping an immunosuppressive environment. The retina is protected by an outer BRB, formed of tight junctions between RPE cells, and an inner BRB, composed of tight junctions between retinal capillary endothelial cells. The BRB regulates the flow of molecules in and out of the retina and keeps noxious and harmful substances outside of the retinal microenvironment preventing also the entrance of circulating immune cells (Cunha-Vaz et al., 2011). Specialized immune cells, namely microglia, are the only resident immune cells in the CNS. Their role is not only limited to immune surveillance, but they play important functions during neuronal development, differentiation and homeostasis (Reichenbach and Bringmann, 2020). During adulthood, immune cells protect neurons from hostile stimuli and are also engaged in neuronal crosstalk and metabolism. Activation of inflammatory responses from intrinsic or extrinsic stimuli in the CNS is primarily designed to the removal of the inflammatory stimulus, but an uncontrolled inflammatory response is associated to neurotoxic effects (Griffin et al., 1987). Microglia has immune surveillance function in the CNS and the retina, being the first inflammatory cells recruited after a noxious stimulus (Jin and Yamashita, 2016; Reichenbach and Bringmann, 2020). Concomitantly with the initiation of the inflammatory response, alternatively activated microglia (called M2) secretes anti-inflammatory cytokines belonging to the interleukin family (e.g., IL-4, IL-10, IL-13, IL-18) and acts in tissue repair (Zhao et al., 2022). Prolonged inflammation, otherwise, leads to classical activation and migration of the microglia (called M1) representing a severe burden to the retinal structure and function, not only for the detrimental effect of activated microglia, but also for the severe alteration in the cytoarchitecture of the retina itself (Martínez-Gil et al., 2022).

### 3.1 Molecular pathways associated to inflammation in retinal degeneration

It is now well established that, during the retinal degenerative process, inflammation is a prominent hallmark which hampers retinal function and worsen photoreceptor survival because the mutation-driven photoreceptor degeneration causes persistent microglia activation (Kaur and Singh, 2022). In RP, genetic insults in rods cause the primary wave of photoreceptor cell death, leading to the release of damage-associated molecular patterns (DAMPs) signals (e.g., ATP, glutamate, HMGB1, HSP, or DNA), that in turn trigger microglia activation (Mahaling et al., 2022; Martínez-Gil et al., 2022). Alongside, stressed photoreceptors expose phosphatidylserine, which induces phagocytosis of mutant cells by the microglia (Zhao et al., 2015). Activated microglia itself secretes several inflammatory cytokines (e.g., TNFα, IL-1β and IL-6) and leads to the production of ROS (as discussed in the previous chapter), increasing photoreceptor damage and ultimately leading to retinal degeneration (Mahaling et al., 2022). The uncontrolled chronic inflammatory state and deprivation of anti-inflammatory factors in the RP retina is detrimental for the highly organized retinal tissue and increases the disorganization of retinal morphology. Specifically, the activation and migration of microglial cells and infiltrating macrophages toward the retinal degenerating outer nuclear layer (ONL, containing photoreceptor cells) alters the finely wired structure of ONL with the inner nuclear layer (INL, containing bipolar cells), amplifying retinal remodeling (Pfeiffer and Jones, 2022). In the healthy retina, resting microglial cells are usually localized in the proximity of the plexiform layers between ONL and INL, where they assume a ramified morphology not affecting synaptic transmission (Reichenbach and Bringmann, 2020). Activated microglia rapidly changes morphology to ameboid-shape with increased cell soma, defined as gliotic response, and starts to invade the photoreceptor layer producing pro-inflammatory cytokines and enhancing cellular damage and degeneration. Among the inflammatory mediators, tumor necrosis factor alpha (TNFα) and its receptors TNFR1 and 2 were found strongly upregulated in RP animal models and also in serum and aqueous humor of RP patients (Appelbaum et al., 2017; Lu et al., 2020; Okita et al., 2020). These cytokines are mainly secreted from activated microglia and they exacerbate photoreceptor degeneration by activating death pathways through receptor-interacting serine/threonine kinase 1/3 (RIP1/3) mediated necroptosis (Huang et al., 2018).

Inflammation during RP is mainly driven by the activation of resident microglia but also involves Müller glia hypertrophy and loosening of the BRB, which allows the entrance of circulating immune cells (Chen et al., 2022). The interplay between microglia and Müller glia cells is crucial for maintaining retinal homeostasis and function. Müller glia cells structurally span the entire thickness of the retina and have a neurotrophic function. Upon prolonged photoreceptor degeneration, Müller glia cells respond with a reactive gliosis, which initially is associated to the release of neuroprotective factors but becomes harmful with time (Goldman, 2014). In fact, gliotic Müller glia has both beneficial and detrimental effects in the RP retina. Hypertrophic Müller glia starts to isolate the site of degenerating photoreceptors by secreting neuroprotective factors (e.g., TGF-β, Midkine, PEDF), helping to reduce photoreceptor degeneration and preserving the correct structure of the surrounding tissue (Bringmann et al., 2006; Chen et al., 2022). The chronic inflammatory microenvironment of neurodegenerative retinopathies induces Müller glia to seal the damaged tissue, leading to the formation of an impenetrable scar in the retina, which also interferes with delivery of neuroprotective drugs (Martínez-Gil et al., 2022; Pfeiffer and Jones, 2022). While the activation of Müller glia is necessary to control infections and recruitment of resident microglia in the retina, the pro-inflammatory stimuli, derived from degenerating rods, induce the upregulation of the chemokine CX3CL1 (C-X3-C motif chemokine ligand 1) and maintain a chronic activation of microglia, thus altering the finely tuned inflammatory cascade (Bringmann et al., 2006; Schmalen et al., 2021; Chen et al., 2022).

### 3.2 Therapeutic approaches to target inflammation

**FIGURE 2:**
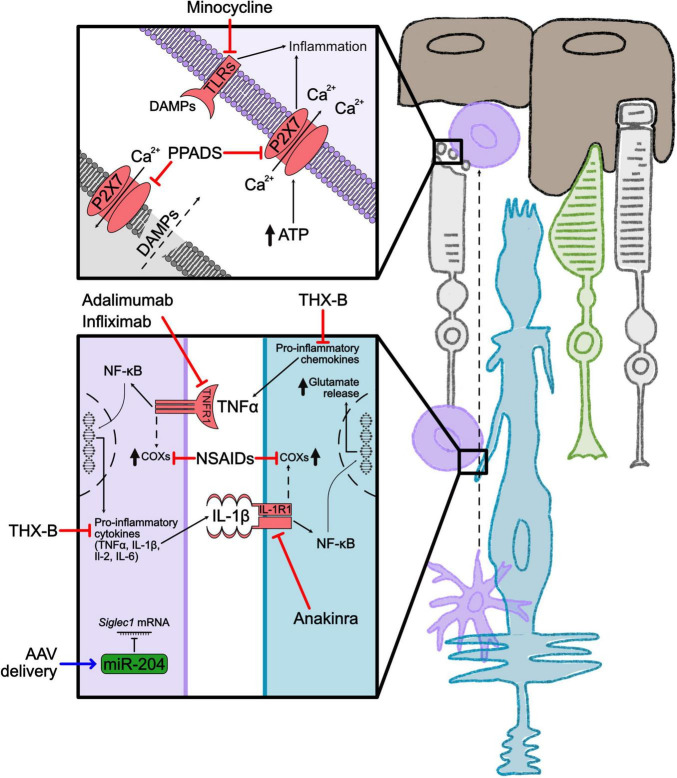
Inflammation in RP and associated treatments. Schematic representation of the main inflammatory cells and cytokines in the degenerating retina. Cones are shown in green, rods in light gray, Müller glia in blue, microglia in violet and RPE in brown. Squares highlight cell-cell interplay in inflammation. Red lines indicate approaches to inhibit inflammatory mediators. The blue line indicates a gene therapy approach. Dashed arrows indicate indirect effects. ATP, adenosine triphosphate; AAV, adeno associated virus; COXs, cyclooxygenases; DAMPs, damage-associated molecular patterns; IL, interleukin; IL1R1, interleukin 1 receptor 1; miR-204, microRNA-204; NF-κB, nuclear factor kappa-light-chain-enhancer of activated B cells; NSAIDs, non-steroidal anti-inflammatory drugs; PPADS, pyridoxal-phosphate-6-azophenyl-2′,4′-disulfonic acid; P2X7, purinergic receptor P2X7; *Siglec1*, *Sialic Acid Binding Ig Like Lectin 1*; THX-B, 1,3-diisopropyl-1-[2-(1,3-dimethyl-2,6-dioxo-1,2,3,6tetrahydropurin-7-yl)-acetyl]-urea; TLRs, toll-like receptors; TNFα, tumor necrosis factor α, TNFR1, tumor necrosis factor receptor 1.

Targeting the inflammatory process as a treatment for RP encountered several drawbacks, because of the dual role of M1 and M2 microglia and of Müller glia cells in tissue repair and in inflammation. The administration of the broad-spectrum immunosuppressant cyclosporine A to RCS (Royal College of Surgeons) rats, a model of RP, hampered retinal functionality and reduced visual acuity (Cooper et al., 2016). Based on this evidence, development of therapies should contemplate, for example, specific targeting of activated microglia or reduction of the pro-inflammatory cytokines secreted in neurodegenerative retinal disorders (Figure 2). These approaches may result as interesting therapeutic options, not only for RP but also for other retinal degenerative pathologies (i.e., age-related macular degeneration and diabetic retinopathy).

One strategy to limit chronic inflammation is to block the signaling cascade derived from the interaction of pro-inflammatory toll-like receptors (TLRs) and their DAMPs ligands. TLR2 and its downstream adaptor protein myeloid differentiation primary response 88 (MyD88) were found upregulated in *rd10* mice and mice expressing the dominant proline 23 to histidine (P23H) mutation in rhodopsin. Genetic knockout of TLR2 showed beneficial effects on the two RP models, providing evidence that interfering with the DAMPs pathway could act as a neuroprotective treatment (Sánchez-Cruz et al., 2021). Minocycline is a potent antibiotic able to block microglia activation in RP. Although the specific mechanism of minocycline in RP is not fully understood, it was found able to block TLR2 and 4 and consequently inhibit the downstream nuclear factor kappa-light-chain-enhancer of activated B cells (NF-kB) and mitogen-activated protein kinase (MAPK) pathways (Pierdomenico et al., 2018). Another strategy tested in the *rd10* mouse model of RP to restrain activation of the TLR pathway was based on the selective block of the downstream adaptor MyD88, for example by interfering with the interaction of MyD88 death domain with interleukin-1 receptor-associated kinase 2/4 (IRAK2/4), which is necessary for the signaling mediated by TLRs (Chen et al., 2020; Garces et al., 2020). Modulation of extracellular DAMPs released from dying photoreceptors was also proven to reduce inflammation in RP models. ATP is released from dying cells as pro-inflammatory signal and is sensed through the purinergic receptor P2X7, exposed also on photoreceptor cells, and promotes Ca^2+^ influx, K^+^ efflux and stimulates the inflammasome assembly (Swanson et al., 2019). Treatment of *rd1* mice with pyridoxal-phosphate-6-azophenyl-2′,4′-disulfonic acid (PPADS), a P2X7 antagonist, could reduce photoreceptor cell death (Puthussery and Fletcher, 2009).

Non-steroidal anti-inflammatory drugs (NSAIDs), like bromfenac, ketorolac, nepafenac, and diclofenac, are already in clinical applications for ocular conditions like glaucoma and age-related macular degeneration (Chen et al., 2021). This class of molecules acts as a broad-spectrum inhibitor of the inflammatory cascade by blocking the activity of COX (cyclooxygenase 1, 2 and 3) enzymes (DuBois et al., 1998). An upregulation of COX1, among other pro-inflammatory markers, was observed in the RP murine model *rd10*. The inhibition of COX1, by the NSAID SC-560, or its genetic ablation in the *rd10* mutant mouse could reduce the release of pro-inflammatory cytokines TNFα and IL-1β, slow photoreceptor degeneration and increase visual acuity (Yang W. et al., 2020).

Tumor necrosis factor alpha (TNFα) is one of the key mediators of inflammation in RP, and it is well documented that the reduced expression of TNFα in mouse models of RP could delay cone photoreceptor degeneration and improve retinal structure (Rana et al., 2017). Pharmacological modulation of the TNFα pathway with adalimumab, which interferes with TNFR1 mediated activation of NF-kB and MAPKs, restrained microglia activation and photoreceptor cell death in the *rd10* mutant retina (Olivares-González et al., 2020). Another inhibitor of TNFα signaling, Infliximab, was found effective in reducing caspase 3 activation and reactive gliosis in cultured porcine retinas treated with Zaprinast, a PDE6 inhibitor (de la Cámara et al., 2014). Treatment with THX-B {1,3-diisopropyl-1-[2-(1,3-dimethyl-2,6-dioxo-1,2,3,6-tetrahydro-purin-7-yl)-acetyl]-urea} was applied as strategy to prevent release of TNFα in the *rd10* retina and in the mouse bearing the proline 347 to serine (P347S) mutation in RHO. In these murine models THX-B could inhibit microglia and Müller glia activation (Platón-Corchado et al., 2017).

IL-1β is another pro-inflammatory cytokine found to be upregulated during retinal degeneration, although photoreceptor cell death appears not to be directly mediated by IL-1β itself since photoreceptors poorly express its receptor IL-1R1 (Charles-Messance et al., 2020). Otherwise, IL-1β stimulates the activation of microglia and Müller glia cells through the pathway of NF-kB, p38, JNKs and MAPKs (Gabay et al., 2010). Müller glia cells respond to IL-1β by releasing glutamate which enhances photoreceptor death by excitotoxicity (Charles-Messance et al., 2020). Administration to the *rd10* mouse of anakinra, an antagonist of IL-1R1, preserved the retina from degeneration and reduced inflammation (Zhao et al., 2015).

Modulation of the inflammatory response in the degenerating retina was also approached by gene therapy with one microRNA (miRNA) involved in microglial maturation. Subretinal injection of an AAV expressing miR-204 (microRNA-204) in the mouse model expressing the P347S mutant RHO, significantly diminished microglial activation. The reduction of expression of *Siglec1* (Sialic Acid Binding Ig Like Lectin 1) mediated by miR-204 preserved photoreceptors and increased electroretinogram (ERG) response (Karali et al., 2020).

In summary, targeting inflammation in inherited retinal degeneration may represent a good option for hindering photoreceptors demise, improving retinal function and maintaining the physiological connections between photoreceptors and glial cells. It is noteworthy that the inflammatory response plays also protective functions on the unhealthy tissue, thus the complete ablation of inflammatory actors can exacerbate the disease. Correct modulation of the inflammatory cascade should be achieved by acting on more than one player of inflammation. In RP, multiple cellular and molecular mechanisms can trigger microglia and Müller glia activation, hence combinatorial treatments need to be evaluated to enhance photoreceptor survival.

## 4 High levels of cGMP

Cyclic guanosine- 3′,5′-monophosphate (cGMP) is a diffusible second messenger synthetized by the enzyme guanylate cyclase (GC). cGMP synthesis and hydrolysis in photoreceptors is tightly regulated by retinal guanylate cyclase (RetGC), guanylate-cyclase activating protein (GCAP), PDE6 enzyme activity and intracellular Ca^2+^ levels ([Ca^2+^]_*i*_). cGMP has a pivotal role in phototransduction by regulating Ca^2+^ influx through the cyclic nucleotide-gated channel (CNGC), and hence controlling the photoreceptor signal transduction. As a second messenger, cGMP also activates protein kinase G (PKG 1 and 2), serine/threonine kinases that act on many targets and cellular functions (Li et al., 2023). The key role played by cGMP in photoreceptors associates its uncontrolled increase to retinal degeneration (Power et al., 2020; Rasmussen et al., 2023).

### 4.1 Molecular pathways associated to cGMP in retinal degeneration

In the photoreceptor outer segment, in dark conditions, cGMP levels are elevated, leading to the activation of CNGC. These channels allow an influx of Na^+^ and Ca^2+^ ions. Ca^2+^ ions are extruded by the Na^+^/Ca^2+^/K^+^ exchanger (NCKX), using the positive extracellular to intracellular Na^+^- K^+^ gradient to remove Ca^2+^ from the intracellular compartment. The Na^+^ excess goes to the photoreceptor inner segment where is removed by the ATP-driven Na^+^/K^+^ exchanger (NKX). This ionic flux generates the so called dark current (Schnetkamp, 1995; Wetzel et al., 1999; Fain et al., 2001). High levels of Ca^2+^ ions inhibit GCAP, the RetGC activator, restraining RetGC activity and, consequently cGMP synthesis. This negative feedback controls cGMP levels that are maintained at physiological range of 1–5 μM in the photoreceptor cell. During light stimulation, photon absorption causes conformational changes in RHO, that leads to the activation of the G-protein transducin, which activates PDE6 and stimulates cGMP hydrolysis. CNGC are closed due to low levels of cGMP and, subsequently, intracellular Na^+^ and Ca^2+^ ions concentrations are reduced. The steady activity of NCKX maintains the membrane hyperpolarized, the electro-chemical signal is not transmitted, and the synaptic glutamate release is inhibited. As feedback loop, low Ca^2+^ levels promote the association of Mg^2+^ to GCAP, switching the protein from an inactive to an active conformational state. As a consequence, GCAP activates RetGC which, in turns, synthesizes cGMP, restoring its intracellular levels and promoting CNGC opening.

**FIGURE 3:**
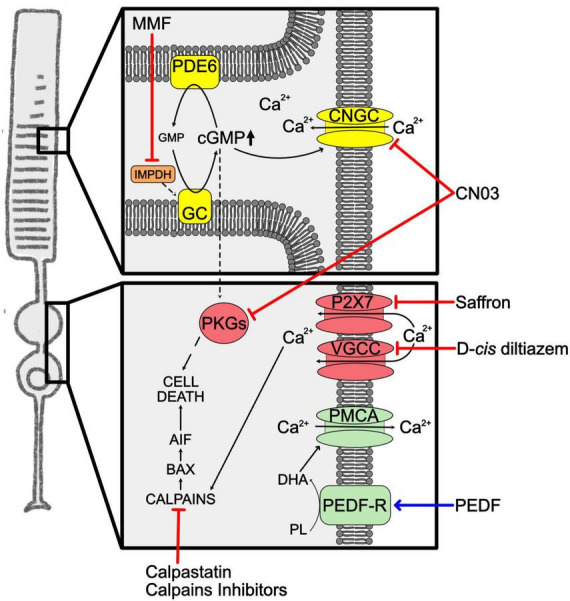
Unbalanced cGMP and calcium in RP. Representation of the increased levels of cGMP and Ca^2+^ in RP rods. Phototransduction cascade is shown in yellow, Ca^2+^ overload mediators are in red, Ca^2+^ clearance mediators in green. Squares highlight cellular compartments where increases of the second messenger occur. The blue arrow indicates overexpression of neuroprotective factors. Red lines indicate approaches to inhibit second messengers related cell death pathway. AIF, apoptosis-inducing factor; BAX, BCL2-associated X protein; cGMP, cyclic guanosine monophosphate; CNGC, cyclic nucleotide-gated channels; CN03, Rp-8-Br-PET-cGMPS; DHA, docosahexaenoic acid; GC, guanylate cyclase; GMP, guanosine monophosphate; IMPDH, inosine-5′-monophosphate dehydrogenase; MMF, mycophenolate mofetil; P2X7, Purinergic receptor P2X7; PDE6, phosphodiesterase 6; PEDF, pigmented epithelium-derived factor; PEDF-R, pigmented epithelium-derived factor-receptor; PKGs, cGMP-dependent protein kinases; PL, phospholipids; PMCA, plasma membrane calcium ATPase; VGCC, voltage–gated calcium channels.

Increased cGMP levels have been correlated to retinal photoreceptor degeneration in murine mutants of the PDE6 enzyme (*rd1* and *rd10*) (Farber and Lolley, 1974; Wang et al., 2018; Das et al., 2021) and in the *Aipl1* (*Aryl hydrocarbon receptor interacting protein like 1*) knockout mouse, that lacks a protein important for the stability of the PDE6 enzyme (Ramamurthy et al., 2004). High levels of this second messenger were reported also in RP mutants of genes not directly involved in cGMP regulation, i.e., *Prph2* (*Peripherin 2*), *Cngb1* (*Cyclic Nucleotide Gated Channel Subunit Beta 1*), *Rho* and *Cnga3* (*Cyclic Nucleotide Gated Channel Subunit alpha 3*) (Arango-Gonzalez et al., 2014; Paquet-Durand et al., 2019b). The toxic effect of high cGMP may be linked to high [Ca^2+^]_*i*_, caused by sustained opening of CNGC, as well as to activation of its main effectors, PKG enzymes (Figure 3). Nevertheless, it is still unclear what are the targets that, once activated by high [Ca^2+^]_*i*_ or phosphorylated by PKGs, mediate photoreceptor cell death. Specifically, high cGMP can cause photoreceptor degeneration through several proposed mechanisms: (1) cGMP opening of CNGC, causing Ca^2+^ overload and activation of calpain proteases that trigger the apoptosis-inducing factor (AIF) (Sanges et al., 2006); (2) cGMP activation of PKG signaling with phosphorylation of yet unknown cell death inducers (Paquet-Durand et al., 2009); (3) cGMP/PKG activation of histone deacetylase (HDAC)/PARP signaling (Sancho-Pelluz et al., 2008). These three mechanisms may promote AIF activation and translocation from mitochondria to the nucleus, chromatin condensation, and subsequent cell death.

### 4.2 Therapeutic approaches to target high cGMP

The imbalance of cGMP levels seems to be an initial event in RP and the estimation that cGMP dysregulation can drive rod cell death in up to 30% of RP led several researchers to develop neuroprotective therapies targeting the cGMP pathway (Das et al., 2021).

Mycophenolate mofetil (MMF) is a drug able to reversibly inhibit inosine monophosphate dehydrogenase (IMPDH) and blocks the *de novo* cGMP production. This enzyme is widely expressed by photoreceptors, so MMF can mainly target the degenerating cells in RP. The daily treatment of *rd1* and *rd10* mice with MMF restrained photoreceptor death and delayed retinal degeneration. In clinical studies, MMF demonstrated to be well tolerated for long periods in patients with organ transplants, uveitis and rheumatoid diseases, favoring the idea of treatment approaches also for RP patients (Yang P. et al., 2020).

The inhibition of cGMP-downstream pathways represents another possible way to reduce intracellular cGMP. An interesting approach was based on developing cGMP analogs able to inhibit PKG and/or CNGC. This idea was based on the knowledge that activation of PKGs is often linked to cell death, as demonstrated on tumor cell lines in which treatment based on PKG-activating cGMP analogs could constrain cell proliferation and promote apoptosis (Hoffmann et al., 2017; Vighi et al., 2018a). Analogs of cGMP can be synthesized with residue substitutions that enable them to inhibit cGMP targets. Specifically, Rp-configurated phosphorothioate modified cGMP with the addition of β-phenyl-1,N^2^-etheno-modification (PET) onto the Rp-cGMPS backbone are cGMP analogs that act as inhibitors (Schwede et al., 2000). Among several cGMP analogs, Rp-8-Br-PET-cGMPS (CN03) was shown to inhibit both PKG and CNGC. The neuroprotective effects of CN03 were confirmed on several retinal degeneration models, i.e., *rd10*, *rd1*, and *rd2*, *in vitro* on retinal explants and *in vivo* using a delivery system based on glutathione-targeted PEGylated liposomes (LP-CN03) able to cross the BRB. The systemic administration of LP-CN03, starting at the onset of the pathology, markedly preserved retinal histology and function (Vighi et al., 2018b). Subsequent studies searched for targets of CN03 using affinity chromatography and mass spectrometry and 7 known cGMP-binding proteins, i.e., PKG1β, PDE1β, PDE1C, PDE6α, and protein kinase A (PKA), as well as other 28 proteins, that included MAPK1/3, were identified (Rasmussen et al., 2023).

A drawback of this treatment is the possible off-target of CN03 on cone CNGC, thus affecting vision mediated by cones. To assess this issue, CN03 was tested in *Xenopus laevis* oocytes expressing CNGCs in combination with another cGMP analog, the 8-pCPT-cGMP, a specific activator of cone CNGC. This experiment confirmed that CN03 preserved cone functionality and, at the same time, normalized rod function in the presence of excessive cGMP (Wucherpfennig et al., 2022).

Taken together, these studies suggest that cGMP analogs acting as inhibitors represent a promising tool in RP treatment, not only for the wide range of mutations that can be targeted, but also for the high specificity of these molecules for their targets linked to retinal degeneration. Nevertheless, to translate this research to the clinic, toxicological studies are required together with evaluation of a possible impact of CN03 on intracellular signaling of cGMP, because fine regulation of the intracellular levels of cGMP are important for the functionality of the retina.

## 5 Calcium overload

Ca^2+^ plays fundamental regulatory functions in photoreceptor cells. Ca^2+^ flows from the extracellular environment and within intracellular organelles, such as mitochondria and ER, to maintain cytosolic Ca^2+^ at nanomolar levels and at 10,000-fold difference with the extracellular environment. This gradient is adjusted by Ca^2+^ channels and Ca^2+^ pumps. As mentioned in the previous chapter, in normal dark conditions cGMP controls the entrance of Ca^2+^ ions through the CNGC in photoreceptor cells.

### 5.1 Molecular pathways associated to calcium overload in retinal degeneration

Pathological increase of cytosolic Ca^2+^ is a consequence of diverse disease-related events concerning genetic defects that affect enzymes regulating cGMP levels. Mitochondria and ER alterations can also affect Ca^2+^ homeostasis in photoreceptor cells. Specifically, Ca^2+^ overloads have been identified as a harmful occurrence at early stages of photoreceptor degeneration (Paquet-Durand et al., 2019a). In fact, increased [Ca^2+^]_*i*_ was detected in animal models of RP caused by mutations in different genes, identifying high intracellular Ca^2+^ as a common mechanism during the photoreceptor degenerative process (Power et al., 2020). Possible mechanisms that cause high [Ca^2+^]_*i*_ have been characterized in retinas from murine models of RP. ER-stress caused by the P23H mutant RHO was correlated to increased intracellular Ca^2+^ (Comitato et al., 2019, 2020). The connection of CNGC activity to high [Ca^2+^]_*i*_ came from double *rd1* and *Cngb1^–/–^* mutant mice. This mutant mouse lacks functional PDE6 enzyme, needed to hydrolyze cGMP, as well as one functional subunit of CNGC, which is needed for Ca^2+^ influx through the cGMP activated channel. The double mutant mice displayed reduce photoreceptor cell death, even in the presence of high cGMP (Paquet-Durand et al., 2011). This study confirmed the key role of CNGC in Ca^2+^ influx in photoreceptor cells and suggested that this channel may, at least in part, mediate increases of [Ca^2+^]_*i*_ when is kept open by uncontrolled levels of cGMP. The homeostasis of [Ca^2+^]_*i*_ in photoreceptor cells is also modulated by the Cav1.4 L-type calcium channels (VGCC), present at the synaptic terminals, and by the P2X7-type purinergic receptor, that regulates Ca^2+^ influx into the inner segment and that we previously mentioned as mediator of the inflammasome assembly. Interestingly, ATP mediated over-activation of the P2X7-type purinergic receptor was associated to high [Ca^2+^]_*i*_ and retinal degeneration.

Molecular studies on RP mutant photoreceptors identified downstream targets of high [Ca^2+^]_*i*_ (Figure 3). Calpain proteases have been identified as targets activated by Ca^2+^ in degenerating retinas. Calpains are cysteine proteases synthesized as inactive pro-enzymes that undergo autoprocessing, an activity that is stimulated by Ca^2+^. Calpains are present in the cell as heterodimers, composed of an 80 kDa catalytic subunit and a 28 kDa regulatory subunit, that are kept at an inactive state in the ER by association with an endogenous calpain inhibitor, called calpastatin. Not only high Ca^2+^ could be linked to calpain activation in the degenerating retina, but also reduced calpastatin has been correlated to photoreceptor degeneration (Paquet-Durand et al., 2006). Calpains do not directly cause cell death, but these proteases cleave apoptotic factors, such as AIF, that induces chromatin fragmentation (Sanges et al., 2006). Downregulation studies of specific calpains identified calpain 1 as the primary mediator of photoreceptor cell death. This same study also defined that calpain 1 triggers the pro-apoptotic BCL2-associated X, apoptosis regulator factor (BAX) in the *rd1* mutant retina (Comitato et al., 2014).

### 5.2 Therapeutic approaches to target high calcium levels

Treatments aimed at the modulation of calpains confirmed their role as effectors of Ca^2+^-induced cell death in RP and demonstrated to be very promising strategies to reduce photoreceptor demise in RP. Several calpain inhibitors were tested *in vitro* and *in vivo* in murine models of RP and, despite their efficacy in granting short term neuroprotection, these studies highlighted possible toxic effects of these drugs upon prolonged administration (Paquet-Durand et al., 2006, 2010; Sanges et al., 2006). Targeting channels for calcium influx in photoreceptors also led to contradictory results. Several attempts based on drugs acting on the VGCC produced controversial data on the neuroprotective properties of VGCC blockers, such as D-*cis* diltiazem, and at the moment VGCC inhibitors attract less interest as therapeutic treatments (Paquet-Durand et al., 2019a). Otherwise, inhibition of the P2X7-type purinergic receptor with saffron provided neuroprotection in *in vitro* cellular models (Notomi et al., 2013; Corso et al., 2016).

A different rationale for developing neuroprotective approaches may be based on boosting calcium pumps favoring the extrusion of Ca^2+^ ions from the photoreceptor cell and prevent calpain activation. The pigment epithelium-derived factor (PEDF) is a glycoprotein preferentially secreted from the apical-lateral side of the RPE toward the photoreceptors, where it acts on photoreceptor morphogenesis, neurite outgrowth and neuroprotection as well as maintains avascularity in this region of the eye (Jablonski et al., 2000; Becerra et al., 2004). Importantly, PEDF levels are altered in eyes affected by retinal degeneration (Ogata et al., 2004). Photoreceptors and ganglion cells of the retina express receptors for PEDF (Aymerich et al., 2001). One of these receptors is PEDF-R, which is a membrane protein with phospholipase activity, localizes at the inner segment of the photoreceptors and mediates activity of PEDF directed to retinal cell survival, as demonstrated *in vitro* and *in vivo* (Subramanian et al., 2013; Kenealey et al., 2015). PEDF was shown to be able to delay photoreceptor degeneration in spontaneous and genetically induced RP models (Cao et al., 2001; Miyazaki et al., 2003; Murakami et al., 2008; Akiyama et al., 2012; Comitato et al., 2018). The neuroprotective activity in the *rd1* mutant retina of PEDF was demonstrated to alter [Ca^2+^]_*i*_ with a decrease of Ca^2+^ ions in photoreceptors cells exposed to PEDF. The mechanism was unraveled *in vivo* by concomitant treatments with different drugs that block specific Ca^2+^ transporters. In this study PEDF was shown to act on plasma membrane Ca^2+^ ATPase (PMCA) pumps to help Ca^2+^ clearance from photoreceptor cells (Comitato et al., 2018). The authors of this study suggested that, based on the knowledge that PEDF-R transduces the PEDF signal by increasing intracellular DHA, DHA may bind PMCA and potentiate its activity, as previously demonstrated in cardiomyocytes (Mączewski et al., 2016).

The above-described studies sustain the view of increased [Ca^2+^]_*i*_ as playing an important role in the photoreceptor degenerating process by the activation of multiple cell death mechanisms. Possible correlation to inflammation was also suggested through the P2X7-type purinergic receptor. New approaches targeting these mechanisms are needed to evaluate long-term effects of such treatments.

## 6 RHO misfolding and ER-stress

**FIGURE 4:**
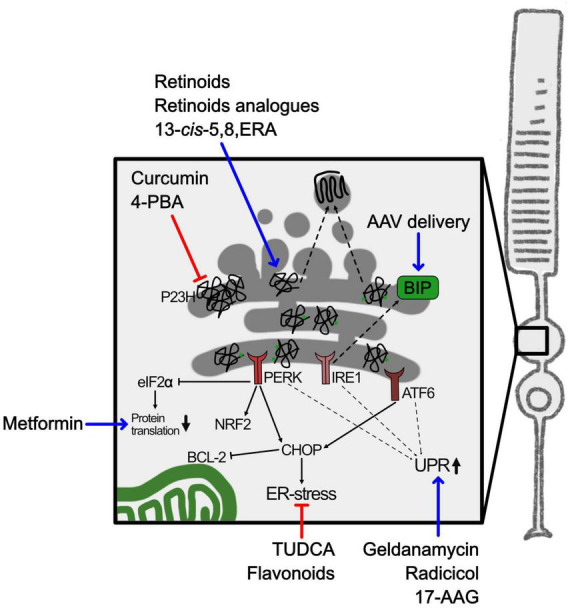
ER-stress and RHO misfolding in RP. Schematic representation of ER-stress caused by misfolded RHO in adRP. The square highlights the inner segment containing the ER. Blue arrows indicate either pharmacological or gene therapy approaches to upregulate chaperons or UPR mediators. Red lines indicate approaches to interfere with aggregation of misfolded proteins. 4-PBA, 4-phenylbutyric acid; 13-*cis*-5,8-ERA, 13-*cis*-5,8-Epoxy Retinoic Acid; 17-AAG, 17-allylamino-17-demethoxy-geldanamycin; AAV, adeno associated virus; ATF6, activation transcription factor 6; BCL-2, B-cell lymphoma 2; BIP, binding immunoglobulin protein; CHOP, C/EBP homologous protein; eIF2α, eukaryotic initiation factor-2α; IRE1, inositol-requiring enzyme 1; NRF2, nuclear factor erythroid-2-related factor 2; PERK, protein kinase RNA-Like ER Kinase; P23H, proline 23 to histidine mutation in rhodopsin; TUDCA, tauroursodeoxycholic acid; UPR, unfolded protein response.

The *RHO* gene is one of the main targets for mutations related to RP, accounting for 30% of autosomal dominant RP (adRP) and 8 to 10% of all RP (Zhen et al., 2023). Around 90% of mutations in *RHO* cause impairment in protein folding, compromising normal protein function, and are usually associated to adRP. RHO is synthesized in the rod photoreceptor cell inner segments and transported to photoreceptor outer segment, where it is densely packed into stacks of membranous disks, and this is a critical step for photoreceptor maturation. RHO binds to 11-*cis* retinal and, upon light stimulation, couples to G protein transducin initiating the phototransduction cascade. The failure of RHO to fold properly, as a consequence of genetic mutations, precludes protein trafficking to the outer segment and RHO is retained and accumulates in the ER, inducing ER-stress (Figure 4; Gorbatyuk et al., 2020).

In an effort to categorize RHO mutants based on the effect of the mutations on protein maturation/function, RHO variants have been divided into the following classes: post Golgi trafficking and impaired outer segment targeting (class 1); ER retention and impairment in retinal binding (class 2); disordered vesicular trafficking and endocytosis (class 3); perturbed post-translational modifications (class 4); altered transducin activation (class 5); constitutive activation (class 6); impaired dimerization (class 7) (Athanasiou et al., 2018). Several studies characterized the molecular outcomes to misfolded mutant rhodopsin in different animal models and led to the proteostatic stress hypothesis for class 2 mutations (Athanasiou et al., 2018). A more detailed classification for the misfolding RHO variants was recently based on predictive *in silico* structural network perturbation and *in vitro* subcellular localization (Behnen et al., 2018; Felline et al., 2021). RHO mutations were subdivided into 4 clusters depending on molecular misfolding. Molecular misfolding was evaluated *in vitro* by analyzing plasma membrane or ER localization of the protein in transfected cells in the absence or in the presence of retinal, as well as *in silico* by analyzing different structural changes of mutant RHO protein when bound to retinal, in term of restoring the native structure. Based on these elements, cluster 1 mutants had the lowest ER retention rates and highest plasma membrane localization. Mutants in cluster 2 exhibited retention in the ER but, when exposed to retinal, membrane localization was restored. Variants in cluster 3 had high ER retention but were, at least partially, able to translocate to the membrane in response to retinal. Cluster 4 mutants had defects in retinal binding with the highest structural perturbation rates, high ER retention and no revert of these phenotypes when exposed to retinal.

### 6.1 Molecular pathways associated to ER-stress in retinal degeneration

Molecular chaperones, such as heat shock proteins (HSP), photoreceptor specific chaperones or co-chaperones, physiologically bind mutated misfolded RHO (Kosmaoglou et al., 2008). HSP are chaperones that can recognize misfolded proteins and interact by binding to exposed hydrophobic regions, with the aim of preventing protein aggregation in the ER and of mediating the heat shock response (HSR). 11-*cis* retinal itself can act as a chaperone for RHO and assists the correct protein folding (Behnen et al., 2018). Accumulation of unfolded proteins activates the UPR to pause protein synthesis, enhance protein folding, and remove misfolded proteins (Read and Schröder, 2021). The inositol-requiring enzyme 1 (IRE1), the activating transcription factor-6 (ATF6), and the protein kinase R-like ER protein kinase (PERK) are sensors at the ER deputed to the quality control of protein synthesis. The three ER sensors regulate expression of chaperones, such as binding immunoglobulin protein (BIP), rest protein synthesis through phosphorylation of eukaryotic initiation factor-2α (eIF2α) or activate apoptotic responses by expression of several effectors such as C/EBP homologous protein (CHOP) that negatively regulates, among others, the anti-apoptotic factor B-cell lymphoma 2 (BCL2) (Hetz, 2012). During retinal degeneration these pathways were found activated (Chan et al., 2016), as shown by the progressive increased expression of CHOP and decreased expression of BIP in photoreceptors with a misfolding mutation in RHO (Lin et al., 2007). BIP plays several roles in the ER because senses misfolded proteins as well as regulates the leakage of Ca^2+^ from the ER, thus helping to maintain ER homeostasis (Krebs et al., 2015). Moreover, ER-stress can also be activated by oxidative stress and ROS since redox homeostasis is central in the processes of protein folding and disulphide bond formation (Plaisance et al., 2016).

### 6.2 Therapeutic approaches to target ER-stress

Targeting ER-stress was evaluated as a treatment to restrain photoreceptor degeneration. Unfortunately, inhibition of the ER-stress sensor PERK had not the expected protecting effect in a retina bearing a dominant misfolding mutation in RHO and, otherwise, reduced visual function, possibly because PERK also regulates an anti-inflammatory response via NRF2 (Athanasiou et al., 2017a; Comitato et al., 2020). Treatment of other RP models with the same inhibitor was reported to reduce phosphorylation of eIF2α but a complete recovery in translation could not be achieved (Starr et al., 2018). Otherwise, overexpression of chaperones was more successful. In fact, AAV mediated delivery of the chaperone BIP in photoreceptor cells of a rat model expressing the P23H mutated RHO modulated the UPR with a reduction of CHOP and preserved photoreceptors and vision (Gorbatyuk et al., 2010).

Based on the previously discussed observations, one possible strategy to slow down photoreceptor degeneration in adRP caused by mutations in RHO might be the development of pharmacological therapies based either on the modulation of the HSR or on the inhibition of mutant protein aggregation by favoring the correct protein folding or promoting the degradation of misfolded proteins (Figure 4). Several attempts aimed at restoring proteostasis, which is crucial for photoreceptors function and viability, were attempted. Defects in proteostasis can be manipulated by administration of drugs to restore physiological protein trafficking in the photoreceptor, taking advantage of pharmacological chaperones and molecular chaperone-inducers. Most of the studies focused on the P23H misfolding mutation in RHO. Mendes and Cheetham (2008) showed that molecular chaperone inducers, such as radicicol, geldanamycin and 17-allylamino-17-demethoxy-geldanamycin (17-AAG), were able to stimulate HSR and reduce P23H mutant RHO aggregation in cell culture. Metformin, a mild inhibitor of protein translation and approved drug for type II diabetes, could promote P23H RHO folding and trafficking *in vitro*. However, despite its positive effect on P23H RHO protein trafficking to the outer segment, metformin did not protect photoreceptors from degeneration. The authors suggested that forcing trafficking of a misfolded protein may increase structural instability of the rod outer segment (Athanasiou et al., 2017b).

Pharmacological chaperons designed to directly target the misfolded proteins attracted a lot of interest. Based on the knowledge that retinal acts as a chaperone for RHO, small molecules binding to the orthosteric binding site of 11-*cis* retinal might be able to shift the protein folding equilibrium toward the native state. Retinoids (11-*cis* and 9-*cis* retinal) or non-isomerizable retinoid analogs (11-*cis*-7-ring and 11-*cis*-6-membered-ring retinal) improved *in vitro* and *in vivo* trafficking and folding of the P23H mutant RHO protein (Kuksa et al., 2002; Saliba et al., 2002; Noorwez et al., 2003; Krebs et al., 2010; Opefi et al., 2013; Pasqualetto et al., 2021). An interesting new chaperone, the retinoid 13-*cis*-5,8 epoxy-retinoic acid (13-*cis*-5,8-ERA), identified by *in silico* screening, showed a 2-fold greater pharmacological chaperone activity compared to 9-*cis* retinal when tested on its ability to transfer mutant RHO from ER to the plasma membrane *in vitro*. 13-*cis*-5,8-ERA chaperone action is likely due to its ability to bind efficiently and reversibly the retinal-binding pocket in the RHO protein, without forming the Schiff Base, and to improve RHO folding (Behnen et al., 2018; Felline et al., 2021).

Other chemical chaperones, not specific for RHO, have been tested. One example is sodium 4-phenylbutyrate (4-PBA), already clinically approved for many diseases, that was able *in vitro* to reduce aggregation of mutant RHO protein and ER-stress associated with misfolded RHO (Jiang et al., 2014; Qiu et al., 2019). 4-PBA also showed improvement of cone opsin trafficking to the outer segment, cone survival and vision in a mouse model of LCA (Li et al., 2016). While 4-PBA was demonstrated *in vivo* to decrease ER-stress and autophagy and increase proteasome activity, two different studies, one in the P23H mutant mouse and one in the P23H mutant rat, provided opposite results on the ability of 4-PBA to protect photoreceptors from cell death and preserve vision (Athanasiou et al., 2018; Qiu et al., 2019). With the aim of benefitting of the anti-aggregating activity on proteins of curcumin, the administration of curcumin to the P23H rat model of RP was proven to ameliorate RHO localization in the outer segment and retina physiology (Vasireddy et al., 2011). Whether the neuroprotective effect of curcumin was mediated also by its antioxidant and anti-inflammatory actions, as discussed above, needs to be further investigated. Another promising chemical chaperone that have been tested in RP was tauroursodeoxycholic acid (TUDCA), which is a component of bear bile and reported to counteract the apoptosis cascade (Boatright et al., 2009). Overall, TUDCA had beneficial effects on many models of retinal degeneration, including RP models, but the mechanism of action is still not well characterized and might be mediated by restraint of ER-stress as well as by its antioxidant action (Athanasiou et al., 2018; Tao et al., 2019).

Finally, flavonoids, such as quercetin and myricetin, that are compounds with antioxidant properties, could modulate cellular stress pathways with beneficial effects in eye-related diseases. Treatment with flavonoids *in vitro* positively modulated the stability of ligand-free RHO, and *in vivo* delayed retinal degeneration by reducing UPR and oxidative stress in mouse models bearing the P23H RHO mutation (Ortega et al., 2019, 2022).

Overall, restoring native conformation of misfolded RHO is an interesting therapeutic research area. The more than 140 different mutations in RHO hamper the development of mutation-directed treatments. The numerous studies characterizing the molecular effects of the different mutations contributed to the classifications of the mutations and are important for the identification of treatments for a wide number of patients. This knowledge will be helpful to design a more accurate treatment for patients that bear selected adRP mutations causing protein misfolding.

## 7 Conclusion

In this paper we reviewed some of the molecular mechanisms underlying one form of retinal degeneration, RP. Photoreceptors have properties that make these cells particularly vulnerable to different insults. In fact, they are post-mitotic cells and cannot be replaced. They are exposed to light that can oxidize proteins and lipids. The phototransduction cascade, that finely tunes two second messengers, cGMP and [Ca^2+^]_*i*_, when disturbed can trigger cell responses that lead to degeneration. Finally, while residing in an immune privileged environment, genetic insults stimulating cell death mechanisms can compromise the BRB and activate damaging inflammatory pathways. The high genetic heterogeneity and complexity of genetic variations in RP activate multiple and concomitant stress responses with highly complex pathogenetic mechanisms. Nevertheless, some common events have been identified and this knowledge is leading to the development of gene-independent therapeutic strategies that can benefit a large cohort of patients.

Reports on the multitude of neuroprotective agents, that have been tested in different animal models, and the characterization of the specific targets of these treatments will open to the possibility of elaborating combined therapies to target multiple pathways. The future development of such types of treatments requires a solid collaboration among ophthalmologists, geneticists and biotechnologists (Wubben et al., 2019).

## Author contributions

AB: Conceptualization, Writing – original draft, Writing – review & editing. EA: Writing – original draft. AS: Writing – original draft. SD: Writing – original draft. VM: Conceptualization, Funding acquisition, Writing – original draft, Writing – review & editing.

## Footnotes

1
https://web.sph.uth.edu/retnet/
